# Revolutionizing pediatric neuroblastoma treatment: unraveling new molecular targets for precision interventions

**DOI:** 10.3389/fcell.2024.1353860

**Published:** 2024-03-27

**Authors:** Min Zheng, Ankush Kumar, Vishakha Sharma, Tapan Behl, Aayush Sehgal, Pranay Wal, Nirmala Vikram Shinde, Bhosale Sachin Kawaduji, Anupriya Kapoor, Md. Khalid Anwer, Monica Gulati, Bairong Shen, Rajeev K. Singla, Simona Gabriela Bungau

**Affiliations:** ^1^ Joint Laboratory of Artificial Intelligence for Critical Care Medicine, Department of Critical Care Medicine and Institutes for Systems Genetics, Frontiers Science Center for Disease-related Molecular Network, West China Hospital, Sichuan University, Chengdu, China; ^2^ Amity School of Pharmaceutical Sciences, Amity University, Mohali, Punjab, India; ^3^ Amity School of Pharmaceutical Sciences, Amity University, Mohali, Punjab, India; ^4^ GHG Khalsa College of Pharmacy, Ludhiana, Punjab, India; ^5^ Pranveer Singh Institute of Technology, Pharmacy, Kanpur, Uttar Pradesh, India; ^6^ SMBT College of Pharmacy, Ghoti Kh, Maharashtra, India; ^7^ School of Pharmaceutical Sciences, Chhatrapati Shahu Ji Maharaj University, Kanpur, Uttar Pradesh, India; ^8^ Department of Pharmaceutics, College of Pharmacy, Prince Sattam Bin Abdulaziz University, Alkharj, Saudi Arabia; ^9^ School of Pharmaceutical Sciences, Lovely Professional University, Phagwara, Punjab, India; ^10^ Australian Research Consortium in Complementary and Integrative Medicine, Faculty of Health, University of Technology Sydney, Ultimo, NSW, Australia; ^11^ Department of Pharmacy, Faculty of Medicine and Pharmacy, University of Oradea, Oradea, Romania; ^12^ Doctoral School of Biomedical Sciences, University of Oradea, Oradea, Romania

**Keywords:** neuroblastoma, molecular targets, immunotherapy, precision interventions, preclinical studies

## Abstract

Neuroblastoma (NB) is the most frequent solid tumor in pediatric cases, contributing to around 15% of childhood cancer-related deaths. The wide-ranging genetic, morphological, and clinical diversity within NB complicates the success of current treatment methods. Acquiring an in-depth understanding of genetic alterations implicated in the development of NB is essential for creating safer and more efficient therapies for this severe condition. Several molecular signatures are being studied as potential targets for developing new treatments for NB patients. In this article, we have examined the molecular factors and genetic irregularities, including those within insulin gene enhancer binding protein 1 (ISL1), dihydropyrimidinase-like 3 (DPYSL3), receptor tyrosine kinase-like orphan receptor 1 (ROR1) and murine double minute 2-tumor protein 53 (MDM2-P53) that play an essential role in the development of NB. A thorough summary of the molecular targeted treatments currently being studied in pre-clinical and clinical trials has been described. Recent studies of immunotherapeutic agents used in NB are also studied in this article. Moreover, we explore potential future directions to discover new targets and treatments to enhance existing therapies and ultimately improve treatment outcomes and survival rates for NB patients.

## 1 Introduction

NB is a cancer that predominantly impacts young children and emerges in nerve tissues. It frequently begins in the adrenal glands above the kidneys but can also develop in nerve tissue along the spine, chest, abdomen, or pelvis (Neuroblastoma, 1931; Qiu and Matthay, 2022; Jacobson et al., 2023). It is one of the most common cancers in infants and is typically found in children under 5 years old (Pudela et al., 2020; Davidoff, 2021; Jha et al., 2023). In the United States, around 700 to 800 children are diagnosed with NB each year, constituting about 6% of childhood cancers. The relative survival rate over 5 years for children under 15 diagnosed with NB is about 82% (Horton, 2022; Mehrvar et al., 2023). NB is classified as embryonic due to its connection to neural crest cells (NCCs) during fetal development (Ben Amar et al., 2022). NCCs are unique cells vital in early embryogenesis, migrating extensively and contributing to various tissues, including the peripheral nervous system, adrenal glands, heart, and face (Pilon, 2021). They essentially serve as building blocks for these critical anatomical features. Substantial advancements have occurred in our understanding of the molecular mechanisms underlying the development and progression of NB (Gomez et al., 2022; Ponzoni et al., 2022). These research endeavors have identified new focal points for potential treatments (Steen et al., 2023). Genome-wide studies, including genome sequencing, have unveiled fundamental genetic changes driving NB growth (Bell, 2010; Kholodenko et al., 2018a; Tonini and Capasso, 2020). In this article, we have summarized novel targets for NB, such as ISL1 (Zhang et al., 2018; Zhang et al., 2019; Li et al., 2021), DPYSL3 (Cheung et al., 2008; Chicco et al., 2023), and ROR1 (Janovská and Bryja, 2017; Quezada and Lopez-Bergami, 2023), along with immunotherapy which is an emerging and promising treatment approach for this disease. Several identified targets are currently being tested as potential treatments for NB patients. This review offers the latest molecular insights regarding the development and progression of NB with a specific focus on genetic alterations/molecular pathways along with clinical management.

Additionally, we aim to offer perspectives on the potential advantages of combination therapy, which involves using inhibitors targeting multiple pathways. One of the prominent molecular features of the NB is MYCN amplification, which is linked to aggressive tumor growth and poor prognosis, along with an increase of chromosome 17q. This oncogene is amplified in 18%–38% of total genes (Aygun and Altungoz, 2019).

Furthermore, it is expected to detect deletion involving chromosomes 1p and 11q and instances of hyperploidy in NB cases (Attiyeh et al., 2005; Bartolucci et al., 2022). Amplification of the MYCN oncogene leads to overexpression of the MYCN protein, which promotes cell growth and proliferation. Anaplastic lymphoma kinase is another oncogene. TrkA (NTRK1) is another factor linked to a favorable prognosis. It is activated by attacking the NGF ligand and promotes cell differentiation, which causes spontaneous regression of NB (Brodeur et al., 2009).

The ongoing incorporation of cutting-edge treatments for individuals with NB into clinical trials and established clinical practices has led to gradual enhancements in patient survival rates (Filippi et al., 2020). Nevertheless, survivors of high-risk neuroblastoma (HRNB) still encounter long-term side effects stemming from their treatment (Veal et al., 2021). Furthermore, there is currently no curative treatment available for most of the approximately 50% of patients who face a relapse after being diagnosed with HRNB. As diagnostic and molecular profiling technologies progress rapidly, researchers are also witnessing the identification of potential targets for treatment (Advani et al., 2022). Molecular targeted therapy for genomic abnormalities and disrupted pathways offers a promising and innovative approach to NB treatment (Jones et al., 2019; Voicu et al., 2023). This approach holds the potential to enhance treatment effectiveness while minimizing adverse effects. In this context, we have explored the clinical scenario regarding NB therapies within the precision medicine framework. Substantial progress has been achieved in understanding the molecular causes of NB, revealing potential therapeutic targets. Genomic studies have identified genetic changes and disrupted pathways in NB growth. These findings may lead to more effective, less toxic treatments.

Authors also delve into their examination in ongoing clinical trials. Furthermore, future directions have been added for enhancing or creating more effective targeted therapies to improve the survival rates of NB patients while minimizing treatment-related side effects.

## 2 Molecular landscape of pediatric neuroblastoma

NB represents the predominant solid tumors outside the cranial cavity during childhood, particularly prevalent within the first year of life (Park et al., 2010; Irwin and Park, 2015). Its distinctive nature is that infants frequently manifest either localized or metastatic forms of the disease, which may undergo spontaneous regression intervention (Tolbert and Matthay, 2018). In contrast, older children can experience disease progression leading to morbidity or mortality despite prolonged and intensive therapeutic interventions (Ahmed et al., 2017). The “International Neuroblastoma Risk Group Staging System (INRGSS)" is a classification system employed in the medical field to stage and categorize NB (Monclair et al., 2009; Sokol and Desai, 2019; Irwin et al., 2021). NB exhibits significant variability, making it crucial to have a systematic approach to determine the disease’s extent and guide treatment choices. There are various known molecular aspects in NB, such as MYCN amplification, ALK mutations, chromosomal abnormalities (Carén et al., 2008), changes in the pattern of DNA methylation, and tumor microenvironment (Tonini and Capasso, 2020; Raieli et al., 2021). Interactions of the tumor microenvironment with stromal cells and blood vessels also play a crucial role in NB progression. Neutrophins are other growth factors involved in developing and maintaining the nervous system, and their dysregulation further promotes tumor growth and survival.

Over 50 years ago, Conrad Waddington introduced the foundational principles of the ‘epigenetic landscape’ to elucidate the fundamental mechanisms governing normal cell differentiation (Baedke, 2013). These studies revealed that transcription factors play pivotal roles in shaping and directing cellular identity and navigating developmental pathways on Waddington’s Hill. Interactions with diverse DNA regulatory elements within a specific epigenetic framework, coupled with chromatin, contribute to the stability of cellular lineages and fates, akin to the depth of grooves on the developmental hill. In a recent analysis, Flavahan and colleagues proposed the notion of aberrant epigenetic resistance and plasticity, attributing these phenomena to dysregulated chromatin regulator activity, remodeling, and DNA methylation as crucial contributors to tumorigenesis (Flavahan et al., 2017; Durinck and Speleman, 2018). Management approaches for asymptomatic individuals classified as low risk, with a projected survival rate exceeding 98%, typically entail either observation or surgical excision alone.

**FIGURE 1:**
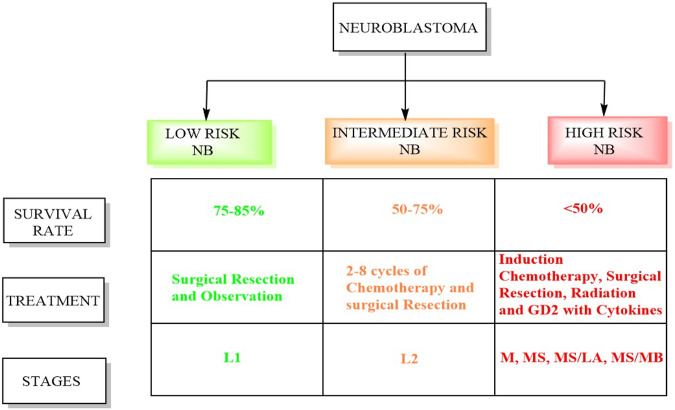
Difference between high-risk and low-risk NB.

Conversely, intermediate-risk patients, characterized by a survival rate exceeding 90%, necessitate moderate doses of chemotherapy tailored to their response alongside surgical resection (Pinto et al., 2015). Patients classified as high risk undergo successive cycles of combination chemotherapy preceding surgical intervention, followed by consolidation therapy involving myeloablative autologous hematopoietic stem cell transplantation and localized radiation therapy (Tolbert and Matthay, 2018). Subsequently, the patients receive immunotherapy and differentiation therapy during the maintenance phase. The INRGSS incorporates tumor characteristics, metastasis, patient age, and specific biological markers (Newman et al., 2019; Campbell et al., 2023). Figure 1 displays the schematic summary between high-risk and low-risk neuroblastoma. Below is a detailed overview of the stages and risk groups in the INRGSS.

- Stage L1: This stage denotes NB confined to its origin point and has not spread to distant sites (Luksch et al., 2016). Tumors in this stage usually exhibit less aggressive biological features, indicating a more favorable prognosis. Patients in stage L1 are considered low-risk and typically have better treatment outcomes (Newman et al., 2019).
- Like stage L1, stage L2 NB is localized but characterized by unfavorable biological traits, suggesting a more aggressive disease. Patients in this stage are classified as intermediate risk, and their treatment may involve more intensive therapies than those in stage L1.
- Stage M indicates that NB has spread or metastasized to distant areas in the body, such as bones, bone marrow, lymph nodes, or other organs. The presence of metastases elevates the risk associated with the disease. Patients in stage M are classified as high-risk and typically require aggressive treatment strategies, including chemotherapy.
- Stage MS represents a subgroup of stage M and includes patients with metastatic NB who exhibit unique clinical features impacting treatment decisions. These circumstances might include patient age, tumor biology, or other relevant factors. Despite these unique features, patients in stage MS remain classified as high risk. The MS/LA subgroup comprises patients with metastatic NB who have a restricted number of metastatic sites. The presence of limited metastases can influence the treatment approach. In stage MS/MB, myc avian myelocytomatosis viral oncogene neuroblastoma (MYCN) amplification is a genetic alteration that increases the tumor’s aggressiveness. This subgroup encompasses patients with metastatic NB who exhibit MYCN amplification.

### 2.1 Comparison between different molecular features in high-risk neuroblastoma (HRNB)

MYCN amplification, ALK mutations, chromosomal abnormalities, changes in the pattern of DNA methylation, and tumor microenvironment are the primary molecular features in HRNBs. MYCN is found in approximately 25% of all NB people with a poor prognosis. It regulates cell proliferation, and its over-expression disrupts cell cycles by inhibiting apoptosis (Rickman et al., 2018). MYCN positively influences the expression of essential cellular regulators, such as E2F and ID2 inhibitors, which regulate cell cycle progression (Woo et al., 2008). In a cohort study by Pugh and colleagues, the authors study the somatic mutations in HRNB on 240 affected people. 9.2% of the total cases comprised of ALK mutation, PTPN11 is expressed in 2.9%, ATRX (2.5%), and MYCN was observed to be 1.7%. Significantly elevated levels of germline variants were observed within the genes ALK, CHEK2, PINK1, and BARD1 (Pugh et al., 2013). Another study by Molenaar et al. showed that chromothripsis and neuritogenesis were two major gene alterations continuously occurring in HRNB. They found 7% cases of ALK mutations, 3% of TIAM1, and 18% of chromothripsis (Molenaar et al., 2012).

## 3 Therapies tailored to specific genetic and molecular alterations in neuroblastoma

### 3.1 Current targeted therapies and novel potential targets in NB

#### 3.1.1 Targeting ISL1

**FIGURE 2:**
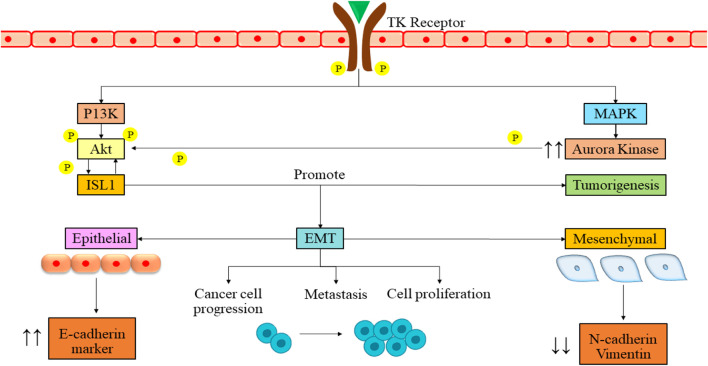
Signaling pathway of ISL1 in NB mediation. The binding of growth factor to the tyrosine kinase receptor leads to the cross-phosphorylation of the receptor. The phosphorylation then leads to activation of PI3K and MAPK pathways. These pathways further lead to the phosphorylation and activation of ISL1. The activation then leads to various cellular processes such as tumorigenesis, cancer cell progression, metastasis, and cell proliferation. PI3K/Akt, phosphatidylinositol 3-kinase/serine/threonine kinase family; EMT, epithelial to mesenchymal transition; ISL1, Insulin gene enhancer binding protein; MAPK, Mitigen-activated protein kinase; TK, Tyrosine kinase; P, Phosphorylation.

ISL1, a transcription factor containing a LIM homeodomain first discovered as a protein that interacts with an enhancer region of the insulin gene, influences its expression (Karlsson et al., 1990). Recent research has highlighted the significant role of ISL1 in cancer progression, primarily attributed to its irregular expression (Agaimy et al., 2013). ISL1 is also associated with triple-negative breast cancer (Zhang et al., 2018), melanoma (Zhang et al., 2018), and gastric cancer (Guo et al., 2019) and has also been found to be a regulator of CD1 and c-Myc genes. ISL1 plays a role in controlling separate temporal gene expression patterns essential for the proliferation and differentiation of sympathetic neurons, either directly or indirectly. ALK, LMO1, and PROX1 are some of the genes modulated by the ISL1 gene, which activates the oncogenic pathways of NB (Cheung et al., 2008; Zhang et al., 2018). ISL1 and GATA3 synergistically activate the signaling pathways by tumor growth and differentiation, which can be a therapeutic target in NB (Zhang et al., 2019). It has prognostic value in gastric and bladder cancers and can serve as a biomarker in NB (Kitchen et al., 2015). ISL1 gene is aurora kinase A (AURKA), a widely distributed protein kinase with crucial functions in cell division (Fulcher and Sapkota, 2020). It is acknowledged to be a potent oncogene and a possible target for cancer (Fadaka et al., 2020; Du et al., 2021). A study also revealed that the ISL1 transcription factor enhances cell proliferation through phosphatidylinositol 3-kinase/protein kinase B (PI3K/AKT) pathway by upregulation of the AURKA enzyme, which is responsible for the survival of NB cells (Figure 2). This study revealed that ISL1 might offer a promising avenue for future therapeutic intervention. They found that PI3K inhibitor LY294002 induces apoptosis in cells. There was a dose-dependent increase in the epithelial marker E-cadherin, along with a corresponding decrease in the levels of mesenchymal markers in Western blot analysis. Notably, metastatic NB cells, SK-N-BE with MYCN amplification and SK-N-SH without MYCN amplification, already express ISL1.

Interestingly, when authors boost ISL1 expression using an overexpression vector, it amplifies the cells’ ability to proliferate and migrate. These findings confirm that ISL1 functions as an oncogene in NB. Finally, they assert that ISL1 triggers epithelial-to-mesenchymal transition (EMT) in NB through PI3K/AKT pathway activation (Li et al., 2021).

#### 3.1.2 DPYSL3 as a potential target

DPYSL3, alternatively labeled as collapsing response mediator protein 4 (CRMP4), represents a human gene responsible for producing a protein termed dihydropyrimidinase-like 3 (Ponnusamy et al., 2014). It is a member of the CRMP family and is involved in various cellular functions, encompassing aspects like neuronal development, guiding axons, and governing microtubule dynamics (Kanda et al., 2014; Ponnusamy et al., 2014). Specifically, DPYSL3 is predominantly found in the nervous system, promoting neurites, guiding axons, and shaping the structural framework of the nervous system (Kawahara et al., 2013; Manivannan et al., 2013). It engages in interactions with various proteins and molecules necessary for these processes. It helps enhance neurite growth, guides axons, and shapes the nervous system’s structural framework (Desprez et al., 2023).

**FIGURE 3:**
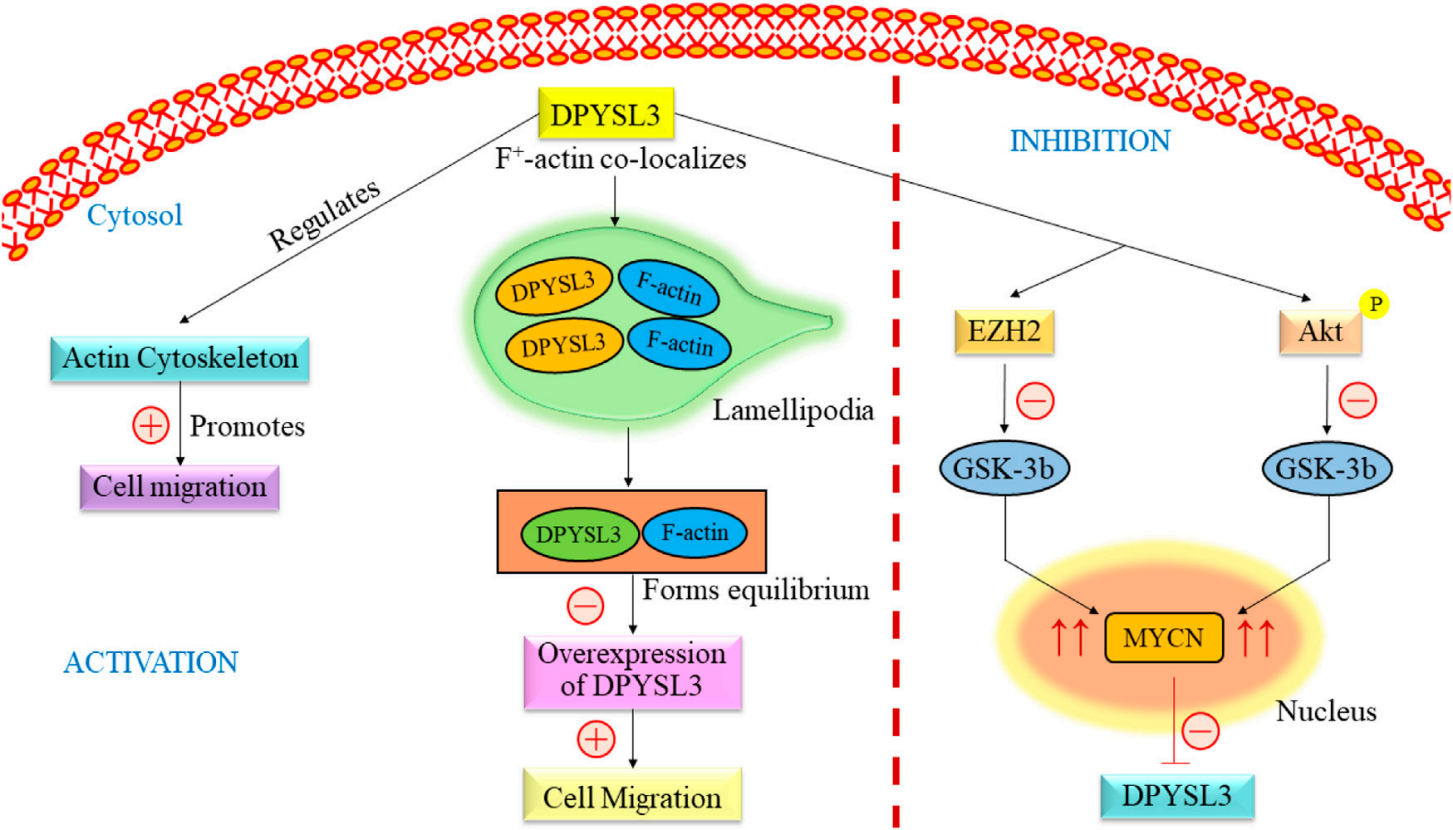
Overview of the signaling pathway of DPYSL3. DPYSL3 is primarily located in the nervous system and is crucial in enhancing neurite growth, guiding axons, and shaping the nervous system’s structural framework. This gene further regulates the actin cytoskeleton, which promotes the cell migration. F-actin co-localizes in lamellipodia and maintains the equilibrium, causing overexpression of DYPSL3 following the promotion of cell migration. The level of DPYSL3 changes with a change in MYCN expression, which GSK-3β regulates. DYPSL3, Dihydropyrimidinase-like 3; EZH2, Enhancer of zeste homolog 2; Akt: Akt serine/threonine kinase family; MYCN, myc avian myelocytomatosis viral oncogene neuroblastoma.

DPYSL3 gene is closely found in the cytosol of NB cells, where it co-localizes with f-actin. The co-localization from a rib-like structure inside the lamellipodia (Figure 3) (Chicco et al., 2023). The primary relation between this gene and F-actin states is an increased level of DPYSL3, which helps migrate B35 NB cells. Conversely, reducing DPYSL3 enhanced cell migration and disrupted lamellipodia (Rosslenbroich et al., 2005; Adam et al., 2020). Studies employing genetic methods have revealed an intriguing relationship between DPYSL3 levels and MYCN expression. It indicates that MYCN inhibits DPYSL3 in NB cells, possibly through an enhancer of zeste homolog 2 (EZH2). GSK3b may also play a role in mediating this negative regulation (Alabed et al., 2010). Furthermore, it is established that AKT phosphorylates and subsequently deactivates GSK-3b in NB cells. Inactivation of GSK-3b is linked to elevated MYCN protein expression (Chicco et al., 2023). Thus, an increase in MYCN level results in the suppression of DPYSL3. Tan and colleagues conducted a study investigating the correlation between DPYSL3 and MYCN in retinoic acid induced cell proliferation and differentiation, further increasing mRNA expression. They found that 72 kDA isoform was unchanged and 62 kDA isoform level increased (Tan et al., 2013). A previous study demonstrated that the 65 kDA form is less phosphorylated and undetected. This change depends on phosphatase regulated by retinoids (Gaetano et al., 1997). DPYSL3 is upregulated by GSK-3B, and their association has already been reported (Alabed et al., 2010; Ong Tone et al., 2010). Consequently, elevated levels of MYCN, either through amplification or due to Akt-mediated GSK-3b inactivation, would suppress DPYSL3 in NB cells (Le Grand et al., 2020; Gao et al., 2024). This mechanistic insight underscores the crucial prognostic significance of DPYSL3 expression (Tan et al., 2013).

#### 3.1.3 MDM2-p53 as potential target

**FIGURE 4:**
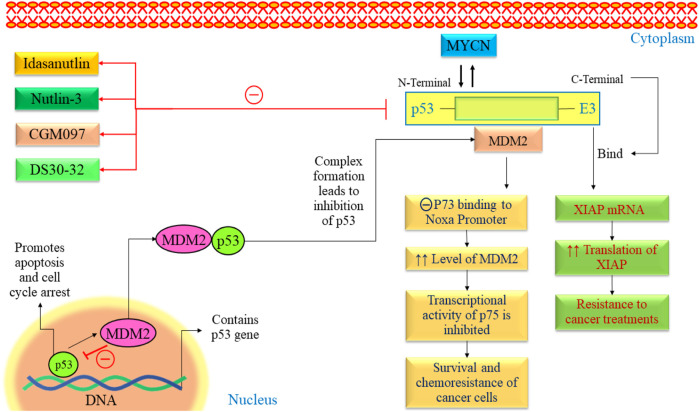
The p53-MDM2 pathway in NB. This pathway is mainly regulated by various proteins such as ARF, BMI-1, BHL, and FAK. All these pathways lead to alteration in cell cycle arrest, proliferation, angiogenesis, and promotion of apoptosis. FAK, Focal Adhesion Kinase; MYCN, myc avian myelocytomatosis viral oncogene neuroblastoma; MDM2, Murine double minute 2; VEGF, Vascular endothelial growth factor; TSLC1, Tumor suppressor in Lung Cancer; p53, Tumor protein p53.

The exact cause of NB remains largely unknown. Recent advances in genetic research, including whole genome sequencing, have revealed mutations, amplifications, and gene rearrangements linked to NB development (Nicolai et al., 2015; Aygun, 2018). Researchers closely examine oncogenes and tumor suppressor genes to understand their crucial role in NB development. The p53 protein protects cells from genome instability and cancer (Balint and Vousden, 2001). P53 mutation is quite rare in NB (Kattner et al., 2019; Tonini and Capasso, 2020). The p53 tumor suppressor responds to deoxyribonucleic acid (DNA) damage by inducing apoptosis or causing cell cycle arrest (Carvajal and Manfredi, 2013). Over half of human cancers have TP53 mutations, often affecting its DNA binding domain and reducing its transcriptional activity (Olivier et al., 2010; Parrales and Iwakuma, 2015). It implies that p53 is experiencing adverse effects through alternative pathways (Figure 4). MDM2 is upregulated in retinoid-induced NB, and irradiation causes DNA damage. In NB, increased p53 activity suggested a potential for inducing apoptosis in these tumors by boosting p53 (Kim and Shohet, 2009). Several investigations have indicated the presence of consistent MDM2 levels in NB cell lines, even in the absence of MDM2 amplification (Cattelani et al., 2008; Cattelani et al., 2013). Elevated MDM2 expression is a recurring observation in NBs, occasionally attributed to single nucleotide polymorphisms (SNPs) in the MDM2 promoter (Cattelani et al., 2013). Moreover, some studies indicate that MYCN contributes to the reduced p53 activity in neuroblastoma by transcriptionally activating MDM2 expression. All these findings suggested MDM2 as a potential therapeutic target in NB.

The MDM2 oncogene is overexpressed in various human cancers, including NB (Hamzehloie et al., 2012). Elevated levels of MDM2 in tumors are associated with a less favorable outlook for individuals with cancer. MDM2 is found to be a negative regulator of p53 and exhibits various oncogenic processes (Nag et al., 2014). It has been found that the N-terminal of MDM2 binds with p53, and C-terminal works as E3 ubiquitin ligase. Many studies have demonstrated that blocking the interaction between p53 and MDM2 using MDM2 antagonists can trigger apoptotic signaling induced by p53 in NB (Van Maerken et al., 2006; Xue et al., 2007). Using medication to inhibit these genes appears to hold potential as a practical treatment approach for NB.

#### 3.1.4 ROR1 as a potential target

The ROR gene family encompasses two members, namely, ROR1 and ROR2, that exhibit a notable degree of evolutionary conservation among diverse organisms, from metazoans to humans (Zhang et al., 2012a; Yu et al., 2016; Dave, 2020; Mukhtar et al., 2020; Behl et al., 2021). These two proteins displayed a substantial amino acid homology of 58% and were effectively replicated in 1992 from the human NB cell line known as SH‐SY5Y. ROR1 and ROR2 serve as single-pass transmembrane receptors characterized by distinct structural attributes in their extracellular regions, including an immunoglobulin (Ig)-like domain, a cysteine-rich domain (CRD) and a kringle domain (KRD) (Masiakowski and Carroll, 1992; Masiakowski and Yancopoulos, 1998). The CRD present in ROR1 and ROR2 shares similarities with the CRD of the frizzled receptors (FZD), and it serves a vital function in facilitating the binding of WNT ligands. Notably, RORs are the only members of the RTK (Receptor Tyrosine Kinase) family that possess a KRD, and this domain has been demonstrated to be vital for the formation of hetero-oligomers between ROR1 and ROR2 (Henry et al., 2015)**.** An exceptionally high percentage of tumors positive for ROR2 were discovered in specific cancer types. These included breast cancer, where 87% of the tumors exhibited ROR2 expression, glioblastoma with over 90% ROR2-positive tumors, and n NB, where 80% of the tumors showed ROR2 presence (Kim et al., 2015). ROR1 prefers expression over ROR2 in B-cell chronic lymphocytic leukemia (B-CLL) cells. Moreover, it maintains constitutive expression on B-CLL cells, even in the face of B-cell activation induced by CD40L and IL-4. ROR1 is associated with high expression in gastric, mRNA, B-CLL, and non-small carcinoma cell lines (Green et al., 2008). In CLL cells, the expression of ROR1 may be regulated by IL-6 through the Stat3 pathway (Li et al., 2010). In one study, the administration of retinoic acid has been shown to enhance the number of cells from their stem-cell stage to a mature neuronal state, acquiring typical neuronal traits such as the inhibition of proliferation. It states that the modulation of ROR1 by retinoic acid could induce differentiation and reduce cancer growth (Mishra et al., 2018).

**FIGURE 5:**
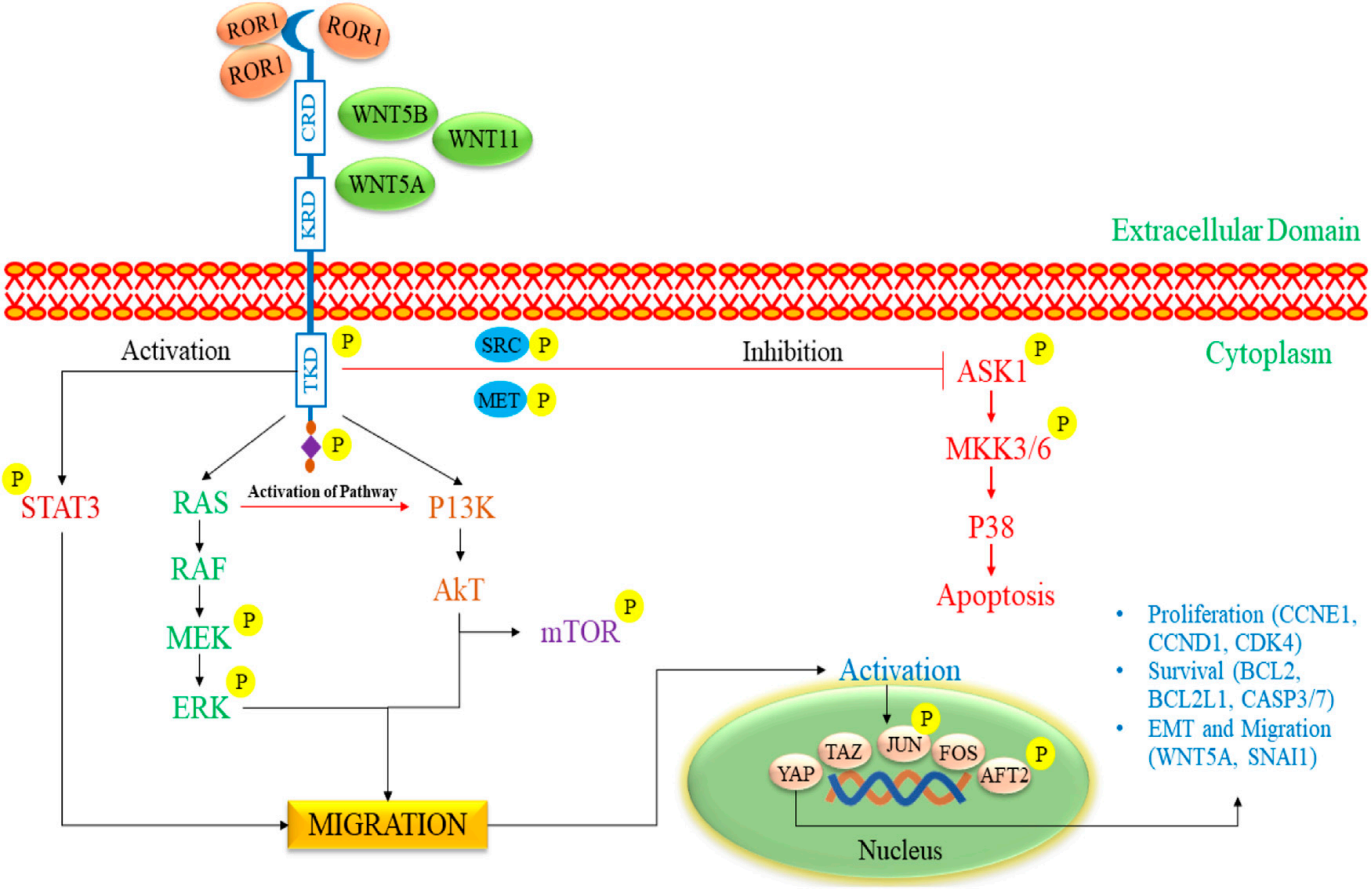
The signaling pathway of ROR1/2 in pediatric NB progression. The initiation of ROR1 signaling commences with the binding of a non-canonical WNT ligand, which triggers the formation of a complex involving either ROR1 and ROR2 or ROR1 and a FZD receptor. It triggers the phosphorylation of ROR1 by various kinases, inhibiting anti-apoptotic pathways and activating pathways such as MAPK/ERK, PI3K/AKT, and NF-κB. These pathways prompt cytoskeletal changes, enhance tumor cell migration, and promote gene expression linked to cell proliferation, survival, EMT, and therapy resistance. ROR1, Receptor tyrosine kinase-like orphan receptor 1; WNT, Wingless/Integrated; FZD, Frizzled receptors; RAS, Rat sarcoma; RAF, Rapidly Accelerated Fibrosarcoma; MEK, mitogen-activated extracellular signal-regulated kinase; ERK, extracellular signal-regulated kinases; mTOR: mammalian target of rapamycin; PI3K, phosphoinositide 3-kinase; Akt, Ak strain transforming; EMT, Epithelial-mesenchymal transitions; ASK1, Apoptosis signal-regulating kinase 1; MKK3/6, MAP kinase 3/6; STAT3, Signal transducer and activator of transcription 3.

The activation of ROR1 signaling commences with the attachment of a non-canonical WNT ligand, which triggers the formation of a complex comprising ROR1 (as shown in Figure 5) (Zhang et al., 2012b). This intricate structure can involve interactions between ROR1 and ROR2 or ROR1 and a FZD receptor. The signaling cascade encompasses the phosphorylation of ROR1 by multiple kinases exerting dual effects (Castro and Lopez-Bergami, 2022). On the one hand, this phosphorylation suppresses anti-apoptotic pathways, while on the other hand, it activates downstream pathways such as MAPK/ERK, PI3K/AKT, and NF-κB. Activating these downstream pathways has various consequences (Menck et al., 2021; Quezada and Lopez-Bergami, 2023). This process may result in cytoskeletal rearrangements correlated with elevated tumor cell migration. Furthermore, it can induce a transcriptional response that leads to the upregulation of genes facilitating cell proliferation, cell survival, EMT, or resistance to therapy (Zhou et al., 2020; Zhang et al., 2021).

### 3.2 Novel therapies (clinical trials) in implementing immunotherapy for pediatric neuroblastoma

NB is a complex cancer type with various subtypes and genetic variations, making it challenging to tailor immunotherapies to each case (Kholodenko et al., 2018b). Children have developing immune systems, which might not respond as effectively to immunotherapies and could lead to more severe side effects (Mackall et al., 2014; Varadé et al., 2021). Limited clinical data for pediatric patients with NB means there is a lack of knowledge regarding the safety and effectiveness of these treatments. Determining the appropriate dosage for children based on age and weight can be complicated, and striking the right balance between potential benefits and risks is crucial. Access to clinical trials for pediatric patients may also be limited, restricting their options for innovative treatments (Fletcher et al., 2018). NB’s tumor microenvironment can suppress immune responses, hindering the efficacy of immunotherapies.

Additionally, NB can employ mechanisms to evade the immune system, making it harder to target the cancer cells effectively (Vanichapol et al., 2018; Veschi et al., 2019). Addressing relapse and resistance, developing combination therapies, and considering the psychological and emotional impact on pediatric patients and their families are also part of the challenge. The cost of immunotherapies and the ethical considerations surrounding their use in pediatric cases further complicate the situation (Unguru et al., 2013; Cabral et al., 2023). Despite these difficulties, ongoing research, collaboration, and a holistic approach offer hope for improved immunotherapy outcomes in the fight against pediatric NB. Table 1 comprises clinical trials of drug therapies and immunotherapeutic agents of the last 5 years.

**TABLE 1:** Clinical trials comprising drug therapies and vaccines with immunotherapeutic agents. NB, Neuroblastoma; HRNB: High-Risk Neuroblastoma; CART, Chimeric Antigen Receptor T cells; PSMA, Prostate-specific membrane antigen; EGFR: Epidermal Growth factor receptor; MTD: maximum tolerated dose; DNA, Deoxyribonucleic acid; IL2, Interleukin 2; NK cells, Natural Killer cells; GM-CSF, granulocyte-macrophage colony-stimulating factor; RXRg, retinoid X receptor gamma.

Sr No.	Identifier	Phase	Status	Agent/Drug	Sample size	Start date	Tested sample	Conditions	Comments
1	NCT05754684	2	Recruiting	Natural killer cell, Dinutuximab beta, Interleukin-2	29	01-01-2022	Blood	Relapsed or refractory NB	Quadruple immunotherapy with NK cells, anti-GD2, IL-2 and GM-CSF and RXRg
2	NCT02573896	1	Active, not recruiting	Dinutuximab. NK cells, Lenalidomide	13	14-01-2019	Blood	Relapsed Refractory NB with expanded NK Cells	This trial determines the MTD of NK combined with Dinutuximab
3	NCT05272371	1	Recruiting	Dinutuximab beta in combination with chemotherapy	20	01-12-2021	Tissue	HRNB	To assess the safety and efficacy of patients treated with dinutuximab beta in combination with chemotherapy
4	NCT04239040	1	Recruiting	GVAX vaccine, Nivolumab, Ipilimumab	26	29-01-2020	Tissue	Relapsed, refractory HRNB, pediatric solid tumor	To check the safety and tolerated dose
5	NCT03635632	1	Recruiting	C7-RGD2, CART cells	94	23-04-2019	Tumor, blood sample	Relapsed and refractory NB	Treat patients with C7R-GD2.CART Cells
6	NCT06057948	2	Recruiting	OPT-821 (QS-21) and β-glucan	94	21-09-2023	Blood	HRNB and metastatic	Research on a vaccine combined with beta-glucan for individuals with NB
7	NCT04936529	2	Recruiting	GM-CSF, OPT-821, β-glucan	264	02-08-2021	Blood NB		The study seeks to assess a combination treatment using a bivalent vaccine, β-glucan, and GM-CSF for high-risk NB patients
8	NCT04049864	1	Recruiting	DNA vaccine, *Salmonella* oral vaccine, Lenalidomide	12	09-01-2019	Tumor sample	Relapsed NB	To test the safety and immune response of a DNA vaccine in relapsed NB patients with post-chemotherapy and stem cell transplantation
9	NCT04239040	1	Recruiting	GVAX vaccine, Nivolumab, Ipilimumab	26	29-01-2023	Tissue collection	Relapsed or refractory NB	This clinical research trial aims to investigate the development and utilization of GVAX when used in conjunction with nivolumab and ipilimumab, as a potential therapeutic approach for NB.
10	NCT05650749	1	Recruiting	GPC2 CAR T cells	30	3-05-2023	Blood	Refractory NB, relapsed NB, HRNB	To determine the MTD of GPC2 CAR T cells
11	NCT05990751	1	Not yet recruiting	GD2 CAR T cells	12	01-01-2024	NA	Relapsed or refractory NB	Targeting Chimeric antigen receptor
12	NCT03721068	1	Recruiting	iC9.GD2.CAR.IL-15 T-cells, Cyclophosphamide, Fludarabine	18	19-02-2019	Tissue	NB, osteosarcoma	To identify the maximum tolerated dose
13	NCT04637503	1,2	Recruiting	GD2, PSMA and CD276 CAR-T cells	100	18-11-2020	Blood	NB	To evaluate the safety and efficacy of 4SCAR-T cell therapy
14	NCT03618381	1	Recruiting	4-1BBζ EGFR806-EGFRt	44	22-02-2019	Blood	NB	EGFR806 CAR T Cell Immunotherapy
15	NCT05562024	1	Recruiting	TAA06 Injection	24	30-12-2022	Tissue	B7-H3-positive Relapsed/Refractory NB	to check the tolerability, safety, and cytokinetic characteristics

In recent years, clinical trials have been conducted to assess innovative strategies for NB treatment (Esposito et al., 2017; Tolbert and Matthay, 2018). These approaches frequently incorporate the granulocyte-macrophage colony-stimulating factor (GM-CSF), which enhances immune activation and antibody-dependent cellular cytotoxicity (ADCC). Numerous current clinical trials explore combination therapies encompassing drugs and newly engineered immunotherapies. Table 1 depicts the ongoing trials on different immunotherapeutic targets in NB using novel combination therapies. NCT05754684 is a phase 2 study to check the safety and efficacy of quadruple immunotherapy with NK cells, IL-2, GM-CSF, and retinoid X receptor gamma (RXRg) with inclusion criteria of creatinine clearance of ≥40 mL/min/1.73 m^2^. The condition for this trial is relapsed and refractory NB. This trial (NCT02573896) is being carried out to check the maximum tolerated dose (MTD) when NK cells combine with Dinutuximab. Dinutuximab is a chimeric antibody against GD2, mainly expressed in NB cells. This trial is performed by using Lenalidomide in combination. Trial number (NCT05272371) describes the study evaluation of Dinutuximab beta in combination with chemotherapy. Another clinical trial (NCT04239040) investigates the development and use of GVAX, a GM-CSF-secreting, autologous NB cell vaccine, combined with nivolumab and ipilimumab as a potential NB treatment. In a clinical trial (NCT03635632), scientists will extract blood samples from the patient. They will then enhance the GD2. C7R T cells by introducing a new gene using a specialized virus called a retroviral vector. The GD2. CAR gene enables the T cells to identify and destroy cancer cells, particularly those that are GD2-positive. Another gene called C7R will also be introduced to these cells to extend their survival. Subsequently, the modified T cells will undergo testing to ensure their ability to target and eliminate GD2-positive cancer cells. A trial NCT06057948, started on 21-09-2023, is carried out to test the treatment of β-glucan with bivalent vaccine. In one study (NCT03721068), the safety and efficacy of IL-15, iCaspase9 is being carried out on patients with relapsed and refractory NB.

## 4 Immunotherapeutic approaches to combat neuroblastoma

The positive outcomes observed in immunotherapy involving anti-GD2 monoclonal antibodies prompt an inquiry into whether NB possesses characteristics that make it susceptible to immune responses (Bhoopathi et al., 2021; Morandi et al., 2021). When discussing the immunogenicity of NB, it is essential to be precise in terminology (Webb et al., 2020; Paraboschi et al., 2021). If we consider “immunogenicity” in the context of cancer, the question arises as to whether NB can activate immune responses.

Despite the absence of evidence for an adaptive immune response, a substantial body of evidence indicates that NB, like many other human cancers, possesses inherent mechanisms designed to elude immune recognition (Cervantes-Villagrana et al., 2020). These mechanisms include decreased MHC class-I expression, suppressive myeloid cells, and the production of inhibitory factors like arginase-2 and TGF-β (Cornel et al., 2020; Dhatchinamoorthy et al., 2021). It enhances an intriguing possibility that the relatively low immune activity within NB may result from a lack of inherent danger signals and the tumor’s active immune evasion mechanisms. This observation offers hope for the potential effectiveness of immunotherapeutic strategies. By developing therapeutic approaches capable of targeting NB cells, scientists have managed to induce a novel immune response in tumors that otherwise appear to escape detection by the natural immune system. These immunotherapies, which utilize synthetic immune recognition, are frequently based on monoclonal antibodies (Carazas et al., 2021; Varadé et al., 2021). Monoclonal antibodies designed to target the disialoganglioside GD2, commonly overexpressed on most NB cells, have brought about a transformative impact on the treatment of NB (Hung and Alice, 2019). They have led to a remarkable increase in event-free survival rates, as high as 20%.

Furthermore, synthetic recognition agents can be further developed from antibody derivatives, including CAR-T cells and antibody-drug conjugates. These innovative therapeutic approaches have demonstrated promising results in pre-clinical and early clinical trials, signifying a significant advancement in immunotherapy. TGF-β regulates cellular activities, including controlling cell growth, proliferation, differentiation, and apoptosis. TGF-β was initially thought to inhibit cancer, but recent studies show it can also promote cancer by impairing NK cell function, leading to increased tumorigenesis, metastasis, and drug resistance, which is a promising target for immunotherapy (Slattery and Gardiner, 2019; Wang et al., 2021; Wienke et al., 2021). Table 2 comprises various immunotherapy approaches to combat NB.

**TABLE 2:** Various immunotherapy approaches to combat neuroblastoma. NB, Neuroblastoma; ADCC, antibody-dependent cell-mediated cytotoxicity; CDC: Complement dependent cytotoxicity; PD1, Programmed Cell Death; PD-L1, Programmed Cell Death Ligand 1; CART Cells: Chimeric Antigen Receptor T cells; CTLA4, Cytotoxic T-lymphocyte associated protein 4; IL1/2, Interleukin 1/2; DNA: Deoxyribonucleic acid.

Neuroblastoma immunotherapy approaches	Description	Examples
Monoclonal antibodies	Administration of antibodies that target specific NB cell surface antigens, often leading to ADCC and CDC	Dinutuximab (Unituxin), Naxitumab
Immune checkpoint inhibitors	Blockade of immune checkpoint molecules like PD-1, PD-L1, or CTLA-4 to enhance the activity of immune cells (e.g., T cells) against NB cells	Nivolumab (Opdivo), Pembrolizumab (Keytruda)
CAR T Cell therapy	Genetic modification of patient’s T cells to express CARs targeting NB antigens, leading to targeted cell killing	GD2-specific CAR T cells
Cytokine therapy	Administration of cytokines (e.g., IL-2, IL-12, IFN-γ) to boost the immune response against NB by enhancing T cell and NK cell activity	Interleukin-2 (Proleukin)
Vaccines	Utilization of NB-specific vaccines (e.g., peptide, dendritic cell, or DNA vaccines) to stimulate the immune system to recognize and attack tumor cells	NBL-004 (Vaccine targeting GD2)
Oncolytic viruses	Use gene-modified viruses that selectively infect and kill NB cells while inducing an immune response against the tumor	Adenovirus-based oncolytic therapy
Immune modulators	Administration of immunomodulatory agents (e.g., immune stimulants or suppressors) to regulate the immune response against NB	Interferon-alpha (IFN- α)
Combination therapies	Integration of multiple immunotherapy approaches to maximize the chances of successful NB treatment	CAR T-cells with checkpoint inhibitors

### 4.1 Monoclonal antibodies

Monoclonal antibodies (mAbs) targeting GD2 effectively respond to high-risk NB cases. However, the outcomes and side effects vary among different kinds of anti-GD2 antibodies (Chan and Chan, 2022). One of these immunotherapies is centered on utilizing mAbs to target GD2, which is found in higher quantities than in a control group in cases of NB. GD2 is a disialoganglioside present on the outer surface of all NB cells (Tibbetts et al., 2022; Machy et al., 2023). Including monoclonal antibodies like anti-GD2 in initial and recurrent treatment strategies has significantly improved survival rates and transformed the outlook for children with HRNB (Anderson et al., 2022). Dinutuximab, a monoclonal antibody that targets GD2 found in neuroblasts, enhances survival when incorporated into the treatment plan. Furthermore, the pairing of dinutuximab with chemotherapy has proven to be highly effective in reversing recurrent disease (FDA, 2015). Monoclonal antibodies targeting GD2 enhance the outlook for HRNB, particularly in young children and older patients who undergo this treatment following multiple previous therapies, typically several months post-diagnosis (Paraboschi et al., 2021). In this study, the authors administered anti-GD2 monoclonal antibodies to infants as part of the immunotherapy protocol initiated during or immediately following induction chemotherapy. A total of 33 HRNB patients, all under 19 months old, were treated with either 3F8 (murine monoclonal antibody, 21 patients) or naxitamab (humanized-3F8, 12 patients) via intravenous infusions lasting between 30 and 90 s. Patients were also provided with analgesics and antihistamines. Two cycles of 3F8 were discontinued, one due to preexisting bradycardia and the other because of asthmatic symptoms. In the case of naxitamab, one patient initially received half the prescribed dose on day 1 due to hypotension, but subsequently received the full recommended dose. Toxicity in infants receiving naxitamab at a higher dosage was comparable to older patients. This is reassuring, as the infant HRNB patients had a high potential for cure (Kushner et al., 2023).

### 4.2 Immune checkpoint inhibitors (ICIs)

ICIs have transformed adult cancer treatment, like lung cancer and melanoma (Wagner and Adams, 2017). This achievement has sparked interest in using ICIs for relapsed and resistant pediatric cancers, with three recent clinical studies assessing their effectiveness (Geoerger et al., 2020a; Geoerger et al., 2020b; Davis et al., 2020). These studies have shown disappointing results, with a low objective response rate in pediatric cancer patients when using programmed cell death protein-1 (PD1) inhibitors like pembrolizumab and nivolumab, as well as programmed cell death ligand (PD-L1) inhibitors like atezolizumab. For pembrolizumab, the objective response rate (ORR) according to RECIST v1.1 criteria for solid tumors was 5.5%. As for nivolumab and atezolizumab, no objective responses were observed among patients with solid tumors (Geoerger et al., 2020b; Davis et al., 2020). Simultaneously, reports from individual cases have demonstrated pediatric patients exhibiting a lasting positive response to immune checkpoint inhibitors, mainly when combined with other cancer-fighting medications (Ehlert et al., 2020).

### 4.3 Chimeric antigen receptor T (CAR T)-Cell therapy

HRNB is a prevalent childhood cancer. While most patients attain remission initially, over 50% relapse due to minimal residual disease, frequently resulting in a fatal outcome (Modak and Cheung, 2010; Tolbert and Matthay, 2018; Shohet et al., 2022). According to a recent research study, antibody therapy combined with cytokines to target minimal residual disease resulted in an approximate 20% increase in these patients’ 5-year overall survival rate (Richards et al., 2018; Yu et al., 2021). Despite this progress, 1/3 of children with cancer still need more effective treatments. Inspired by successful CAR T-cell therapy for blood malignancies, several CAR T-cell treatments are now developing for NB patients (Maude et al., 2014; Maude et al., 2018). It is less effective for solid tumors like NB than leukemia, as seen in initial GD2-targeted CAR T trials for relapsed NB patients (Louis et al., 2011; Heczey et al., 2017; Heczey et al., 2020; Straathof et al., 2020). Of 42 patients with active disease across four trials treated with 14.18 or K666 GD2-CAR T-cells, only three experienced prolonged objective responses (Straathof et al., 2020). The glypican 2 (GPC2) antigen is present during the initial stages of fetal development but becomes mostly inactive in normal tissue after that (Bosse et al., 2017; Li et al., 2017; Li et al., 2021; Heitzeneder et al., 2022; Tian et al., 2022). GPC2, found in NB, is a potential immunotherapy target with much lower expression levels than GD2 and B7H3 (Heitzeneder et al., 2022). It has consequences for the CAR’s efficiency because reduced antigen density is linked to decreased CAR interaction, activation, and its ability to combat tumors (Majzner et al., 2020). So, Sun and co-workers developed this therapy for children with NB. They optimized pre-clinical CAR by using interactive engineering to enhance the antitumor effect. The research indicated that among three GPC2-CAR constructs, anti-GPC2 CT3 with a CD28 hinge, CD28 transmembrane, and 4-1BB co-stimulatory domain demonstrated the most effective pre-clinical activity against NB. The authors compared the CT3.28H.BBζ chimeric antigen receptor (CAR) antitumor effectiveness to a GD2 CAR recently undergoing clinical trials with a similar CAR structure (Sun et al., 2023).

### 4.4 Cytokine therapy

Cytokines activate the immune system against tumors and are a promising approach in NB immunotherapy (Pistoia et al., 2011; Cavalli et al., 2020; Yu et al., 2021; Dowsey, 2023). Combining an antibody targeting GD2 (hu14.18) with interleukin-2 (IL-2) has demonstrated significant potential in pre-clinical and clinical settings for treating NB. The therapeutic benefit of IL-2 remains uncertain due to severe toxicities at higher doses and unproven efficacy at lower doses. The authors connected IL15 and IL21 to hu14.18, resulting in improved antibody-dependent cell-mediated killing in immune-competent NB models compared to hu14.18-IL2 (Nguyen et al., 2022). Migration inhibitory factor (MIF) is a versatile cytokine that plays a significant role in various diseases, including cancer. Pre-clinical and clinical research in patients with NB consistently shows that MIF possesses characteristics that promote tumor growth in NB. Levels of MIF are elevated in NB tumor tissues and cell lines, contributing to the increased aggressiveness of NB and assisting in immune evasion (Cavalli et al., 2020).

### 4.5 Vaccines

Cancer vaccines differ from infectious disease vaccines as they are typically therapeutic rather than preventive (Huebener et al., 2003; Bleeke et al., 2009; Komorowski et al., 2018). Only two Food Drug and Administration (FDA) approved preventive cancer vaccines exist for HPV and HBV-related cancers. Challenges in predicting antigens and achieving substantial immune responses impede the clinical progress of anticancer preventive vaccines (Ahmed, 2022). Top-notch cancer immunotherapy aims to harness the immune system’s potential to eradicate cancer cells efficiently. Cancer vaccines face challenges in precisely delivering antigenic markers and adjuvants to coordinate an effective immune response. Messenger ribonucleic acid vaccines show promise in cancer treatment by prompting antigen expression in antigen-presenting cells, leading to adaptive immune responses (Webb et al., 2020; Wolfson et al., 2021).

## 5 Conclusion

In conclusion, the comprehensive review of NB underscores the significance of diverse therapeutic targets, including ISL1, DPYSL3, MDM2-p53, and ROR1. The exploration of these targets has provided valuable insights into potential avenues for interventions in this cancer. The outcomes of clinical trials and other therapies hold evidence for advancing precision medicine. Identifying additional valuable targets and creating more efficient therapies are imperative. Furthermore, delving into novel combinations that incorporate inhibitors addressing multiple targets in conjunction with conventional treatments remains a paramount focus for future research. This direction is poised to advance treatment protocols, enhance results, and extend the survival of high-risk NB in children. Although significant breakthroughs have been achieved in molecularly targeted therapy for NB, applying these findings to clinical disease management has had limited success in previous years. Thus far, the sole approved treatment for pediatric patients with relapsed or refractory HRNB is anti-GD2 monoclonal antibodies. The disparity between research conducted in pre-clinical settings and its translation into clinical trials is striking. There is also a need for novel combinations to target multiple molecular targets in conjunction with traditional therapies.

## 6 Future perspectives

NB, a childhood cancer, is characterized by abnormal development and typically has a low mutational burden, distinguishing it from most adult malignancies. Conversely, drug testing primarily relies on conventional NB cell and mouse models. There is a need to progress towards developing new disease models by harnessing cutting-edge technologies, like 3D tissue-engineered systems and patient-derived xenograft model systems. Researchers must focus on *in vitro* and *in vivo* testing studies to develop new synthetic drugs to treat NB. Further research must be carried out on designing novel inhibitors and immunotherapy. Apart from discovering novel targets for immunotherapy and designing therapeutic interventions, researchers must also prioritize designing a combination of new small NB inhibitors with radiotherapy to inhibit the various signaling pathways. Decreasing the high dose of radiochemotherpeutic agents could be another significant approach to combat NB without toxicity and side effects. New target-based inhibitors are being used for the patients. In summary, the future of immunotherapy and novel targeted therapies in NB treatment is marked by a commitment to innovation, personalization, and collaboration. With ongoing research and technological advancements, people can expect improved outcomes and quality of life for NB patients in the years to come.

## Abbreviations

AKT, Ak strain transforming; CAR T, Chimeric antigen receptor T; CRD, Cysteine-rich domain; CRMP, Collapsin response mediator protein; DPYSL3, Dihydropyrimidinase-related protein 3; DNA, Deoxyribonucleic Acid; EMT, Epithelial-mesenchymal transitions; FDA, Food and Drug Administration; FZD, Frizzled receptors; HRNBL, High-risk neuroblastoma; ISL1, ISL LIM Homeobox 1; KRD, Kringle domain; MAPK, Mitogen-activated protein kinase; MDM2, Murine double minute 2; MIF, Migration inhibitory factor; MYCN, myc avian myelocytomatosis viral oncogene neuroblastoma derived homolog; NB, Neuroblastoma; NCC, Neural crest cells; PI3K, phosphoinositide 3-kinase; ROR1, Receptor tyrosine kinase-like orphan receptor 1; RTK, Receptor Tyrosine Kinase; mAbs, Monoclonal antibodies, PD-L1, Programmed Cell Death Ligand; ORR, Objective response rate; TP53, Tumor protein p53.
